# Activity of DNA polymerase κ across the genome in human fibroblasts

**DOI:** 10.1073/pnas.2403130121

**Published:** 2024-07-01

**Authors:** Mariela C. Torres, Abbey Rebok, Dongxiao Sun, Thomas E. Spratt

**Affiliations:** ^a^Department of Biochemistry and Molecular Biology, Pennsylvania State University, Hershey, PA 17033; ^b^Department of Pharmacology, Pennsylvania State University, Hershey, PA 17033

**Keywords:** DNA polymerase kappa, DNA replication, DNA

## Abstract

DNA polymerase κ (Polκ) is a specialized polymerase that has multiple cellular roles such as translesion DNA synthesis, replication of repetitive sequences, and nucleotide excision repair. We have developed a method for capturing DNA synthesized by Polκ utilizing a Polκ–specific substrate, *N*^2^-(4-ethynylbenzyl)-2′-deoxyguanosine (EBndG). After shearing of the DNA into 200 to 500 bp lengths, the EBndG-containing DNA was covalently bound to biotin using the Cu(I)-catalyzed alkyne–azide cycloaddition reaction and isolated with streptavidin beads. Isolated DNA was then ligated to adaptors, followed by PCR amplification and next-generation sequencing to generate genome-wide repair maps. We have termed this method polymerase κ sequencing. Here, we present the human genome maps for Polκ activity in an undamaged cell line. We found that Polκ activity was enhanced in GC-rich regions, euchromatin regions, the promoter of genes, and in DNA that is replicated early in the S phase.

DNA polymerase κ (Polκ) is one of the 16 DNA-dependent DNA polymerases expressed in humans. Polκ is typically described as a translesion DNA synthesis (TLS) polymerase because of its ability to bypass bulky *N*^2^-alkyl-dG adducts (1, 2). Polκ has also been linked with global genomic nucleotide excision repair (GG-NER) (3), cross-link repair (4), the production of short oligodeoxynucleotides in the ataxia telangiectasia and Rad3-related (ATR) kinase activation pathway (5), and replication of repetitive DNA sequences in undamaged cells (6, 7). An obstacle with determining when and where a specific DNA polymerase is active is that all polymerases utilize undamaged DNA and the four deoxynucleotide triphosphates as substrates.

To examine the role of Polκ, we utilized *N*^2^-(4-ethynylbenzyl)-2′-deoxyguanosine (EBndG, Fig. 1*A*), a nucleoside that has high selectivity for Polκ (8). The ethynyl group on EBndG was utilized to capture and map DNA synthesized with Polκ in a process we termed polymerase κ sequencing (PolK-seq).

**Fig. 1.**
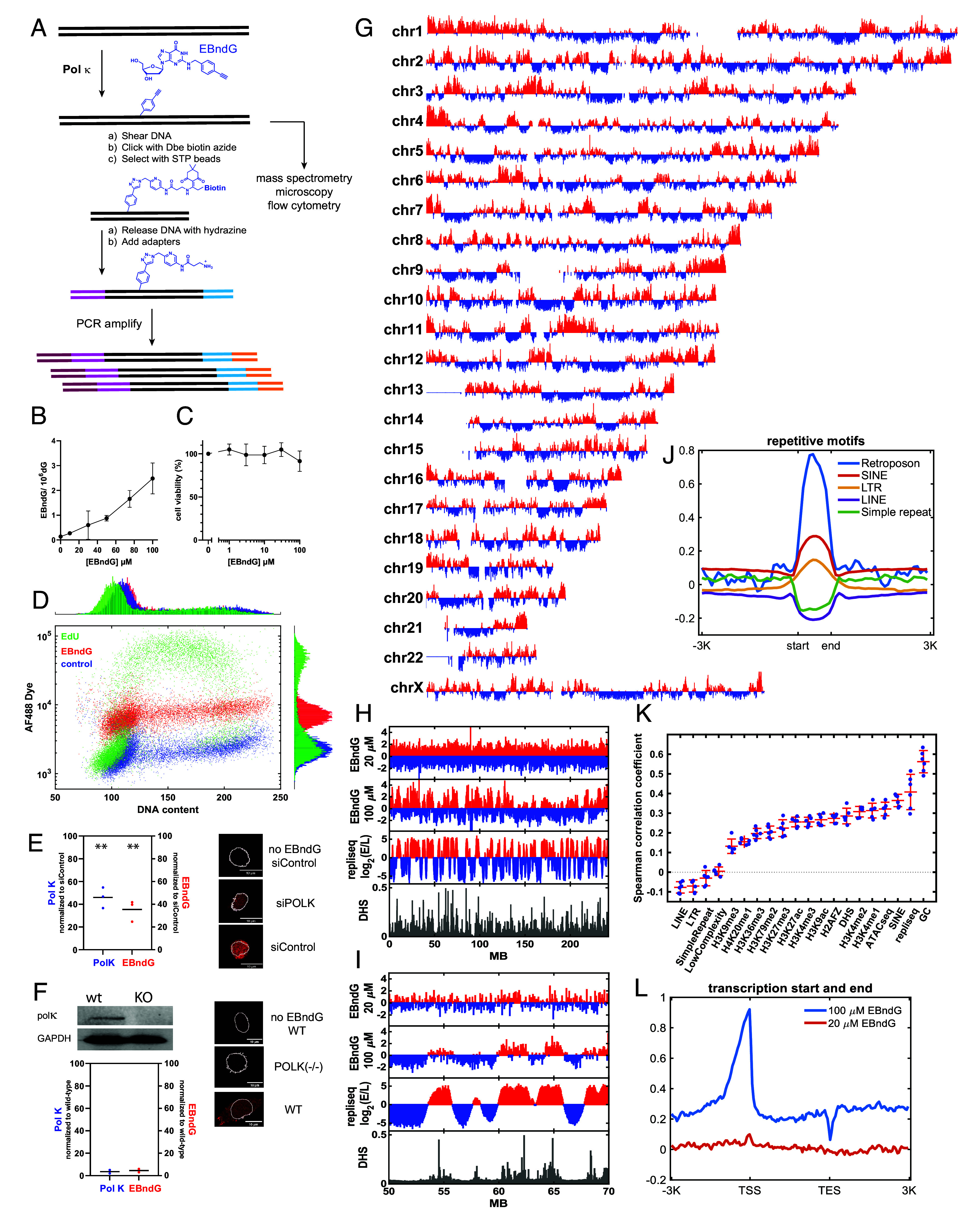
(*A*) Protocol for the preparation of next-generation sequencing libraries. (*B*) Incorporation of EBndG into DNA of GM12878 cells. Data are the mean of two experiments (±SD). (*C*) Cell viability after 72 h EBndG incubation. Data are mean (±SD) of three replicates. (*D*) Flow cytometric analysis of the incorporation of EdU (50 μM) and EBndG (100 μM) into GM12878 cells over 4 h. DNA content was determined by PI fluorescence while nucleoside incorporation was fluorescence of the AF488Dye-nucleoside conjugate. (*E*) Percent of Polκ protein levels (Pol K) and activity (EBndG) in siPolK vs. control siRNA treated cells. Microscopy images show AF594Dye incorporation with no EBndG as well as cells treated with siPOLK and siControl. **P < 0.01 for decreased protein and EBndG vs. siControl. The extent of the nucleus is highlighted by the white lines. (*F*) Relative EBndG incorporation in wild-type HeLa and two clones of POLK(−/−) cells. Microscopy images show representative wt and KO cells, and western blots show Polκ and GAPDH levels. (*G*) Genome-wide map of EBndG in cells exposed to 100 μM EBndG. The *Y*-axis is log2 (EBndG/control) calculated in 50 kb bins across six biological replicates. (*H*) Detailed profile of chromosome 2 in which the EBndG distributed determined at 20 and 100 μM is compared with the replication timing score and the DNase hypersensitive sites. (*I*) Chromosome 2 is examined from 50 to 70 MB. The red vs. blue colors emphasize increased vs. decreased EBndG incorporation as well as early vs. late replication timing. (*J*) Profile of EBndG incorporation against repetitive elements. (*K*) Spearman’s correlation coefficients of relative abundance of EBndG and genomic features in cells exposed to 100 μM EBndG. (*L*) Profiles of EBndG incorporation against transcription start (TSS) and cleavage and polyadenylation (TES) sites.

## Results

### EBndG Incorporation.

DNA was isolated from GM12878 cells treated with EBndG for 4 h. The EBndG incorporation was analyzed by high performance liquid chromatography with mass spectrometry detection and found to be concentration dependent (Fig. 1*B*). EBndG was nontoxic up to 100 μM over 72 h (Fig. 1*C*). Flow cytometry analysis (Fig. 1*D*) showed that EdU was extensively incorporated during the S phase, while EBndG was incorporated into DNA at a much lower level, but the incorporation was cell cycle independent. In addition, EBndG did not alter the cell cycle. EBndG incorporation was dependent on Polκ protein levels, as siRNA knockdown cells showed decreased incorporation of EBndG in proportion to Polκ protein levels (Fig. 1*E*). Furthermore, Polκ-knockout HeLa cells incorporated minimal levels of EBndG (Fig. 1*F*). While Polκ is responsible for the vast majority of the EBndG incorporation, a small fraction of the EBndG may be incorporated by another polymerase.

### PolK-seq.

GM12878 cells were exposed to EBndG for 4 h. After treatment, DNA was isolated, fragmented, and strands containing EBndG were covalently bound to biotin (Fig. 1*A*). Following isolation of the EBndG-containing strands with streptavidin magnetic beads, next-generation sequencing libraries were prepared, and sequences were analyzed using paired-end 50 nucleotide reads. The reads were mapped to the human genome, binned into 50 kb regions, normalized to the total read count, and the difference between EBndG treated and untreated cells determined (Fig. 1*G*). As shown in Fig. 1 *G*–*I*, 100 μM EBndG produced genomic regions that differed in the extent of EBndG incorporation, while at 20 μM, less EBndG was incorporated into DNA (Fig. 1*B*), and we were unable to observe sequence-specific incorporation.

### Comparisons of EBndG with Genomic Data.

We compared our data with previously analyzed DNase hypersensitivity sites (DHS), histone epigenetic marks, repetitive sequences, and replication timing. Results were binned into 50 kb regions, and Spearman’s correlation coefficients were obtained over six EBndG biological replicates (Fig. 1*K*). The experiments with the highest correlation to the EBndG incorporation were the guanine/cytosine (GC) content, early replication timing, DHS, and assay for transposase-accessible chromatin with sequencing (ATAC-seq) regions that are associated with open DNA, as well as the short-interspersed nuclear element (SINE) repetitive sequences. Small inverse correlations were associated with the long interspersed nuclear element (LINE), long-terminal repeat retrotransposons (LTP), and low-complexity repetitive sequences.

The high correlation of EBndG incorporation with GC-rich regions may represent the activity of Polκ on difficult-to-replicate repetitive DNA sequences (6, 9) or the insertion of EBndG opposite cytosine. The correlation between DHS and EBndG incorporation is visualized in Fig. 1 *H* and *I*.

EBndG incorporation was more highly correlated with histone modifications associated with active than with inactive chromatin. The H3K4me1 and H3K4me2 modifications, which are associated with primed enhancers and active genes, respectively, had R values of 0.31 ± 0.03, while the heterochromatin-associated H3K9me3 had R = 0.13 ± 0.03. The two major repetitive sequences showed opposite correlations: the SINEs (R = 0.36 ± 0.03) were positively associated with Polκ-activity, while the LINEs (R = −0.08 ± 0.03) were inversely correlated. These correlations were supported in the sequence analyses in which the EBndG incorporation was compared with the start and end of the repetitive elements throughout the genome in Fig. 1*J*. Notably, the SINE regions had increased EBndG incorporation, while the opposite was observed for LINEs and simple repeats.

We then compared EBndG incorporation vs. DNA replication timing, in which the replication score is log_2_(E/L), where E/L is the ratio of BrdU incorporation in the early vs. late S phase. The incorporation of EBndG correlated with early replication times with R = 0.41 ± 0.09. This association was evident when examining replication timing vs. EBndG incorporation in Fig. 1 *H* and *I*. Since we found that EBndG is incorporated in all phases of the cell cycle (Fig. 1*D*), this result may reflect the propensity for open DNA to be replicated early.

To analyze the influence of gene transcription on EBndG incorporation, we examined the extent of incorporation over the transcription start and polyadenylation sites across the genome. As shown in Fig. 1*K*, cells treated with 100 μM EBndG exhibited elevated Polκ activity in the promoter regions of genes in comparison to the gene body and 3′ regions.

## Discussion

The most studied activity of Polκ is TLS that typically occurs in the late S phase (1, 2). Since euchromatin is repaired more rapidly than heterochromatin (10), we expected heterochromatin to contain increased levels of endogenous DNA damage. In addition, many repetitive DNA sequences are located in heterochromatin (6, 9), which are replicated late in the S phase (11). Consequently, we expected that EBndG incorporation would predominate in heterochromatin and in late replicating sequences. However, using PolK-seq, we found the opposite result: Polκ is more active in regions of the genome that are replicated early in the S phase, open regions of the genome, as well as gene promoters.

The increased Polκ activity in open DNA vs. heterochromatin and early vs. late replication sequences indicates that Polκ has a significant cellular function(s) that is distinct from S-phase TLS. Two possible activities that have been studied are GG-NER (3, 12, 13) and cross-link repair (4). As the incorporation of EBndG into promoter regions of genes (Fig. 1*L*) is not consistent with these results, there may be other cellular processes in which pol κ plays a role.

## Materials and Methods

GM12878 human lymphocytes were treated with 0 to 100 μM EBndG for 4 h. DNA was isolated, sheared and DNA strands containing EBndG were covalently bound to Dde biotin picolyl azide with the Cu(I)-catalyzed alkyne–azide cycloaddition reaction. Next generation sequencing libraries were prepared, and sequences were analyzed with Illumina NovaSeq-6000 using paired-end 50 nucleotide reads. Processed sequencing data have been deposited in the NCBI Gene Expression Omnibus (GEO) (https://www.ncbi.nlm.nih.gov/geo/) under accession no. GSE260451. The code used for data analysis is available at https://github.com/thomas-spratt/POLK-seq. All extended methods are included in *SI Appendix*.

## Supplementary Material

Appendix 01 (PDF)

## Data Availability

Next generation sequencing data have been deposited in Gene Expression Omnibus under accession number (14). All other data are included in the manuscript and/or *SI Appendix*.
