# Supplementary material for: Activity of DNA polymerase κ across the genome in human fibroblasts
Source: Proc Natl Acad Sci U S A. 2024 Jul 1;121(28):e2403130121. doi: 10.1073/pnas.2403130121 (PMC11252913; doi:10.1073/pnas.2403130121)
Supplement: Supplementary file 1 — Appendix 01 (PDF) [file pnas.2403130121.sapp.pdf]

PNAS

Supporting Information for

Activity of DNA polymerase κ across the genome in human fibroblasts

Mariela C. Torres, Abbey Rebok, Dongxiao Sun and Thomas E. Spratt.

* **Corresponding author:** Thomas E Spratt, **Email:** tes13@psu.edu

This pdf file includes:

Extended methods

Materials and Methods

Reagents

AFDye 488 picolyl azide, AFDye 594 and Dde biotin picolyl azide were purchased from Click Chemistry Tools (Scottsdale, AZ). DNA Isolation Midi-Prep Plus and DNA Clean and Concentrator Kits were purchased from Zymo Research. Fetal bovine serum (FBS) was purchased from Biowest (Bradenton, FL). Dynabeads MyOne Streptavidin C1 beads and RNAiMAX were purchased from ThermoFisher. Transfection medium (sc-108062) and UltraCruz transfection reagent (sc-395739) were purchased from Santa Cruz Biotech. DinB CRISPR/Cas9 plasmid (sc-405052), siDinB (sc-60538) and control siRNA were purchased from Santa Cruz Biotechnology (Santa Cruz, CA). Antibodies were purchased from Santa Cruz Biotechnology (DinB sc-166667), and Invitrogen (anti-GAPDH, 39-8600 and anti-mouse-HRP, 31430). N^2-4-Ethynylbenzyl-2'-deoxyguanosine (EBndG) was synthesized as described (1).

Cell Culture

GM12878 human lymphocytes (ATCC-NIST-8398), an ENCODE project Tier 1 human lymphocyte cell line (2), were cultured in Roswell Park Memorial Institute (RPMI) 1640 media supplemented with 15% FBS, 1% penicillin-streptomycin and 2 mM GlutaMax (Corning). The cells were grown at 37 °C with 5% CO_2 at ~80% relative humidity. They were seeded at 300,000 cells/mL and were split when they reached 10^6 cells/mL. The day before experiments, cells were seeded at 500,000 cells/mL and incubated overnight. HeLa cells were cultured in DMEM containing 10% FBS and 1% penicillin-streptomycin. Cells were treated with either 20 μM EdU for two hours or EBndG for four hours, harvested, washed with phosphate buffered saline (PBS) and pelleted. Cells used for DNA isolation were stored at -20°C until use.

Flow Cytometry

Cells were treated with 50 μM EdU for 2h or 100 μM EBndG for 4 h, then harvested by centrifugation (5 min at 500×g). The cells were (a) washed with PBS, (b) treated with 4% paraformaldehyde (PFA) in PBS for 15 min at room temperature, (c) washed with 1% bovine serum albumin (BSA) in PBS (w/v) and (d) treated with 1 mL 0.5% saponin in PBS (v/v) for 10 min. Following saponin treatment, the cells were incubated with the Click Reagent (1 mM $CuSO_4$, 5 μM AFDye 488 Picolyl Azide, 10 mM sodium ascorbate in PBS) for 30 min at room temperature. The cells were washed twice with 0.5% saponin in PBS (v/v) and treated with propidium iodide (PI)-RNase A reagent (75 μM PI, 10 μg/mL RNase A in 1% BSA/PBS). Finally, the cells were analyzed using a BD LSR Fortessa flow cytometer.

siRNA-mediated transfection

GM12878 cells were transfected with 40 nM siDinB and RNAiMAX in antibiotic-free RPMI containing 20% Opti-MEM. After 24 h, cells were pelleted and resuspended in a fresh transfection mixture for an additional 24 h. Following the second transfection, cells were incubated with 100 μM EBndG for 2 h and either plated on poly-D-lysine coated coverslips and PFA fixed or harvested for Western blot protein analysis.

Microscopy

Cells were incubated with 0 or 100 μM EBndG for 2h, fixed with PFA, permeabilized (10 mM Tris-HCl pH 7.6, 150 mM NaCl, 0.5% Triton-X 100) for 20 min, washed with PBS, and clicked as described above with 0.2 μM AFDye 594 picolyl azide for 1.5 h, PBS washed, and rocked for 1 h with 5% BSA/PBS to remove unbound fluorophore. Coverslips were mounted with DAPI fluoromount and imaged using a Leica SP8 STED 3X confocal microscope. Images were analyzed by CellProfiler 4.2.1 to obtain EBndG integrated intensity values. The integrated intensity values were subtracted from the mean values of the no-nucleotide control experiment.

CRISPR knockout

HeLa cells were grown to 70% confluency in a 6-well plate. DinB CRISPR/Cas9 plasmid (10 μL at 0.1 μg / μL) was combined with 140 μL plasmid transfection medium and incubated for 5 min at room temperature. UltraCruz transfection reagent (10 μL) was combined with 140 μL plasmid transfection medium for 5 min. Both solutions were combined and incubated for 20 min. The 300 μL transfection solution was added dropwise to cells in 1 mL fresh DMEM containing 10% FBS. After 24 hours, cells were harvested and two GFP+ cells were sorted into 96-well plates with the BD FACSAria (BD Biosciences, San Jose, CA). Cells were grown and pol κ status was confirmed by western blot.

Western Blot

Cells were lysed (50 mM Tris-HCl pH 7.5, 20 mM NaCl, 10 mM $MgCl_2$, 0.1% SDS) on ice for 30 min with 1X protease inhibitors. Samples were centrifuged at 12,000×g for 10 mins to pellet the debris and supernatant protein concentration was determined using a Nanodrop spectrophotometer. Laemmli buffer was added to samples at a final concentration of 1X and heated at 95 °C for 5 min. Samples were run on a 12% SDS-PAGE gel, transferred to a 0.45 μm PVDF membrane using the Trans-Blot Turbo system, and blocked for 1 h in 5% BSA in 20 mM Tris-HCl, pH 7.6, 137 mM NaCl, 0.1% Tween-20 (TBST). Anti-DinB (1:1000 dilution) or anti-GAPDH (1:5000 dilution) was added and incubated for 2 h at room temperature with rocking. Blots were washed three-times with TBST and incubated with anti-mouse-HRP for 2 h with rocking. Blots were washed three-times, and bands were visualized with ECL substrate (Thermo, 32106) with the ChemiDoc MP imaging system.

CellTiter-Glo Proliferation Assay

Cells were seeded in a 96-well plate at 2,000 cells in 100μL media/well and treated with different concentrations of EBndG for 3 days. CellTiter-Glo 2.0 (Promega) was added at 40 μL/well. Plates were rocked for 1 minute and allowed to equilibrate for 10 minutes. Luminescence values were assessed using the GloMax Navigator Microplate Luminometer (Promega). Luminescence values were corrected with a negative control containing no cells.

DNA isolation

Cells (3×10^7) were suspended in 20 mL 10 mM Tris-HCl, pH 7.4, 0.5 % SDS, 1 mM EDTA, 150 mM NaCl with 2 mg proteinase K and heated at 55° C for 2h. The solution was cooled and treated with one volume of phenol/$CHCl_3$/iso-amyl alcohol (25/24/1). Following vigorous shaking, the layers were separated by centrifugation. The aqueous layer was extracted an additional time and the DNA precipitated by addition of 2 mL 3 M sodium acetate (pH 5.2) and 15 mL isopropanol. The DNA was resuspended in 10 mL Tris-HCl (pH 7.4), 1 mM EDTA and treated with 50 μg RNase A for 1 h at 37°C, followed by 100 μg proteinase K for 1 h at 55°C. The aqueous layer was extracted once with phenol/$CHCl_3$/iso-amyl alcohol followed by a $CHCl_3$ extraction. DNA was precipitated with 1/10 th volume 3 M sodium acetate and 2.5 volumes of ethanol.

EBndG incorporation analysis by HPLC MS/MS

The DNA was redissolved in 10 mM Tris-HCl, pH 8, 10 mM $MgCl_2$, 5 mM $CaCl_2$, and mixed with DNase I (1 unit), phosphodiesterase I (0.005 units) and alkaline phosphatase (10 units) and incubated for 16 h at 37°C. After incubation, 25 μL of hydrolysate was taken for the analysis of dG by HPLC with UV detection. The remaining hydrolysate was loaded on a Strata-X cartridge (30 mg, Phenomenex) activated with 1 mL MeOH and 1 mL H_2O. The cartridge was washed with 1 mL H_2O, 1 mL of 20% MeOH, after which EBndG was eluted with 2 mL of 90% MeOH. The 90% MeOH fraction was concentrated to dryness in a rotary evaporator and redissolved in 50% MeOH. The samples were analyzed using a Sciex QTRAP 6500+ mass spectrometry coupled with a Sciex EXion UHPLC separation system. A 1.7 μm Acquity UPLC BEH C18 analytical column (2.1 x 100 mm, Waters, Ireland) was used to separate EBndG from other impurities. The mobile phase consisted of (A) 0.1 % acetic acid in water, and (B) acetonitrile. The gradient elution was conducted using a flow rate of 0.3

mL/min with a linear gradient from 10% B to 100% B over 3.6 min, followed by holding at 100% B for 1.4 min.

The Sciex QTrap 6500+ mass spectrometer was equipped with an electrospray ionization probe operated in positive mode. The decluster potential (DP) was 57 V, the entrance potential (EP) was 10 V, the collision energy (CE) was 24.7 V, and the collision cell exit potential (CXP) was 15 V, while the curtain gas (CUR) was 35 L/h, the collision gas (CAD) was medium, the ionspray voltage was 5000 V, the temperature was 250 °C, gas 1 was 20 L/h, and gas 2 was 20 L/h. The multiple reaction monitoring mode (MRM) was used to analyze and quantify EBndG, with the transitions of *m/z* 382 $[M + H]^+$ > 266 $[M – deoxyribose + H]^+$. The peak was integrated and quantified by Sciex OS 3.0 software.

At the dose and time point of our study, EBndG was incorporated at a level of 2.5 EBndG per million dG. This level is approximately 1/10th the level of N^2-butyl-2'-deoxyguanosine observed in HEK cells (3).

DNA fragmentation and NGS

DNA (20 μg) in 130 μL 10mM Tris-HCl, pH 8.0 was fragmented using a Covaris E220 focused ultrasonicator in a microTUBE AFA Fiber Snap-Cap (Covaris, 520045) with the Duty Factor set to 10 and Peak Incident Power set to 175W at 200 cycles per burst for 180 seconds per sample. The average size of the DNA was 200 base pairs as determined by agarose gel electrophoresis.

The sheared DNA was reacted with 1mM $CuSO_4$, 2mM tris(benzyltriazolylmethyl)amine (THPTA), 40μM Dde biotin picolyl azide, and 5mM sodium ascorbate in PBS for 60 min with gentle rotation at room temperature. The DNA was separated from the biotin reagents with the Zymo DNA Clean and Concentrator-25 kit, with elution into 10 mM Tris-HCl (pH 8.0), 0.1 mM EDTA. Five percent of each DNA sample was kept aside as an input control.

MyOne Streptavidin C1 magnetic beads were used to capture the biotinated DNA and hydrazine was used to release the DNA. To prepare the beads for DNA binding, 10 µL of the DynaBeads were washed with Tween-20 (0.1 %) in PBS followed by 10 mM Tris-HCl (pH 8.0), 1 M NaCl twice. The beads were suspended in 50 μL 10 mM Tris, pH 8, 2 M NaCl followed by 50 μL of the DNA solution. The suspension was incubated for 1 h, washed twice with 10 mM Tris-HCl (pH 8.0), 1 M NaCl, followed by 10 mM Tris-HCl (pH 8.0) twice. The DNA was released from the beads with 50 μL 2% hydrazine (w/v) in 10 mM Tris-HCl (pH 8.0). The DNA was isolated with a Zymo DNA Clean and Concentrator-5 kit, with elution into 25 μL 10 mM Tris-HCl, pH 8. The DNA yield was 3-10 ng as determined by fluorometric analysis using a Qubit dsDNA high sensitivity assay kit.

The libraries were prepared using the NEB Ultra II DNA Library Prep Kit for Illumina. The samples were submitted to the Penn State College of Medicine Genome Sciences Facility. NGS was performed on a Novaseq Illumina instrument with a 50 cycle paired-end protocol. We obtained over 78 million reads for each sample, which were aligned to the reference human genome (hg38), and >94% reads were successfully mapped to the reference with < 5 % PCR duplicates.

Data analysis

The FASTQ files were mapped onto the hg38 genome with Bowtie2 (4). Proper pairs were selected and duplicates were removed with Samtools (5). The reads were binned with Deeptools multiBamSummary (7). The data were normalized, the difference between the sample (EBndG = 20, 100 μM) and control (EBndG = 0 μM) obtained and plotted with Matlab.

We downloaded the data sets from the ENCODE portal (https://www.encodeproject.org/) (2) with the following identifiers

ENCFF023LTU (H3K27ac), ENCFF131ZGZ (H3K79me2), ENCFF283LNH (H3K4me2), ENCFF291DHI (H3K27me3), ENCFF320OGZ (H3K4me3), ENCFF321BVG (H3K4me1), ENCFF377OJG (H2AFZ), (H4K20me1), ENCFF470YYO (ATAC-seq), ENCFF725UFY (H3K9me3), ENCFF759OLD (DNase-seq), ENCFF981JOU (H3K9ac). The bed files for the repetitive sequences were extracted from 4DNI6. The data from replication timing was downloaded from 4D Nucleome Data Portal (https://data.4dnucleome.org/) (8) with the identifiers, 4DNFI6TILWWX, 4DNFIIMJQ8NT, 4DNFIS7J9B9X, 4DNFIT26294Y.

.

References

1. A. S. P. Gowda, M. Lee, T. E. Spratt, N^2-Substituted 2′-deoxyguanosine triphosphate derivatives as selective substrates for human DNA polymerase κ. *Angew. Chem. Int. Ed.* **56**, 2628-2631 (2017).
2. E. P. Consortium, An integrated encyclopedia of DNA elements in the human genome. *Nature* **489**, 57-74 (2012).
3. Y. Tan *et al.*, DNA Polymerase η Promotes the Transcriptional Bypass of N(2)-Alkyl-2'-deoxyguanosine Adducts in Human Cells. *J Am Chem Soc* **143**, 16197-16205 (2021).
4. B. Langmead, S. L. Salzberg, Fast gapped-read alignment with Bowtie 2. *Nat Methods* **9**, 357 - 359 (2012).
5. H. Li *et al.*, The sequence alignment/map format and SAMtools. *Bioinformatics* **25**, 2078-2079 (2009).
6. A. R. Quinlan, I. M. Hall, BEDTools: a flexible suite of utilities for comparing genomic features. *Bioinformatics* **26**, 841-842 (2010).
7. F. Ramírez *et al.*, deepTools2: a next generation web server for deep-sequencing data analysis. *Nucleic Acids Res* **44**, W160-165 (2016).
8. S. B. Reiff *et al.*, The 4D Nucleome Data Portal as a resource for searching and visualizing curated nucleomics data. *Nature Communications* **13**, 2365 (2022).
